# Changes in Rats’ Gut Microbiota Composition Caused by Induced Chronic Myocardial Infarction Lead to Depression-Like Behavior

**DOI:** 10.3389/fmicb.2021.641084

**Published:** 2022-04-14

**Authors:** Qianwen Wang, Xi Wang, Yong Lv, Chun Yang, Chenliang Zhou, Long Wang

**Affiliations:** ^1^Department of Anesthesiology, Renmin Hospital of Wuhan University, Wuhan, China; ^2^Department of Cardiology, Renmin Hospital of Wuhan University, Wuhan, China; ^3^Cardiovascular Research Institute, Wuhan University, Wuhan, China; ^4^Hubei Key Laboratory of Cardiology, Wuhan, China; ^5^Department of Anesthesiology, The First Affiliated Hospital of Nanjing Medical University, Nanjing, China; ^6^Department of Critical Care Medicine, Renmin Hospital of Wuhan University, Wuhan, China

**Keywords:** chronic myocardial infarction, gut microbiota, depression-like behavior, microbiome-gut-brain axis, hierarchical cluster analysis

## Abstract

Depression is common among patients who have chronic myocardial infarction (CMI). Despite their frequency, depression and CMI are bidirectional related conditions, each is a risk for the other, and they often co-exist, suggesting shared or interacting pathomechanisms. Accumulating data revealed the effects of gut microbiota in terms of regulating depression *via* the gut–brain axis. Thus, we investigated the role of gut microbial dysbiosis in CMI-induced depression-like behavior. Hierarchical cluster analysis of sucrose preference test (SPT) results was adopted to classify the CMI rats into depression-like behavior (CMI + Dep) or non-depression-like behavior (CMI + Non-Dep) phenotypes. First, 16S ribosomal RNA sequencing analysis showed both β-diversity and relative abundance of several gut bacteria significantly differed between the CMI + Dep and CMI + Non-Dep rats. Next, transplantation of fecal microbiota from CMI + Dep rats visibly altered the relative abundance of gut microbiota and also induced depression-like behavior in the antibiotics-treated pseudo-germ-free rats. In conclusion, these findings suggested that dysbiosis in gut microbial composition contributed to the onset of CMI-induced depression-like behavior and that exogenous regulation of gut microbiota composition could be a potential therapeutic strategy for CMI and related depression-like behavior.

## Introduction

With the optimization of evidence-based treatments in modern society, the mortality of myocardial infarction significantly decreased (Saar et al., 2015; Gabet et al., 2016). However, a pile of literature revealed that myocardial infarction survivors were more prone to psychological disorders, such as depression (van Dijk et al., 2016). Over the past 20 years, it has been found that depressed chronic myocardial infarction (CMI) patients have a high risk of cardiac independent risk factors (Brborović et al., 2009) and poor prognosis of further adverse cardiac events (Smolderen et al., 2017). In addition, the post-CMI depression usually reduces the quality of life, increases in stress and exhaustion, and often precipitates hospitalization of the patient. A Global Burden of Disease study launched by the World Health Organization predicted coronary heart disease (including CMI) and depression would be two of the three most disabling disorders worldwide by 2030 (Mathers and Loncar, 2006). Unfortunately, the precise neurobiological mechanisms underlying this correlation of CMI with depression have not been fully described (Grippo and Johnson, 2009). And there is no effective treatment strategy available which can treat both CMI and depression.

Recently, gut microbiota is confirmed to strongly affect brain function and the cardiovascular system (Koopman et al., 2017). Bidirectional microbiota–gut–brain communication has mostly been explored in animal models (Koopman et al., 2017; Lach et al., 2018). Gut microbiota features influenced the host quality of life and depression (Valles-Colomer et al., 2019). Furthermore, gut microbiota translocation was observed in CMI patients (Zhou et al., 2018; Tang et al., 2019). Based on these findings, we speculated that depression caused by CMI may be related to dysbiosis in gut microbiota composition.

In the classical model of depression, behavioral changes of rats included anhedonia, behavioral despair, and psychomotor retardation (Hu et al., 2017). Anhedonia is a central feature of depression, which leads to reduced reward responsiveness, often in the form of reduced sucrose intake or preference. Therefore, for the evaluation of depression-like behavior, the sugar water preference test (SPT) must be selected. In forced swimming test (FST), immobility time could reflect the degree of behavioral despair in rats (Yankelevitch-Yahav et al., 2015). It should be noted that each behavioral change represents only one symptom in rats. In order to evaluate depression-like behavior more accurately and scientifically, we chose SPT and FST to evaluate depression-like behavior in rats. After hierarchical cluster analysis of the results of SPT, CMI group rats were divided into CMI + Dep group and CMI + Non-Dep group in our study. Considering the potential role of gut microbiota in CMI-induced depression, we used 16S rRNA gene sequencing to compare gut bacterial composition between CMI + Dep and CMI + Non-Dep phenotypes. Moreover, we examined the effects of fecal bacteria transplantation from CMI-induced Dep and Non-Dep phenotypes on depressive symptoms and gut microbiota composition of host pseudo-germ-free mice.

## Materials and Methods

### Animals

One hundred male SD rats (age, 6–8 weeks) were purchased from Hunan SJA Laboratory Animal Co., Ltd., (Hunan, China). Animals were housed under controlled temperature (22 ± 2°C), controlled relative humidity (60 ± 5%), and a 12-h/12-h light/dark cycle with *ad libitum* access to food and water. Animals were allowed to acclimate for 7 days before experiments. All experimental protocols and animal handling procedures were carried out in strict accordance with the recommendations in the Guide for the Care and Use of Laboratory Animals, published by the National Institutes of Health (NIH Publications No. 80–23, revised in 1996). This study was ethically approved by the Animal Care and Use Committee of Renmin Hospital of Wuhan University (Wuhan, China).

**FIGURE 1:**
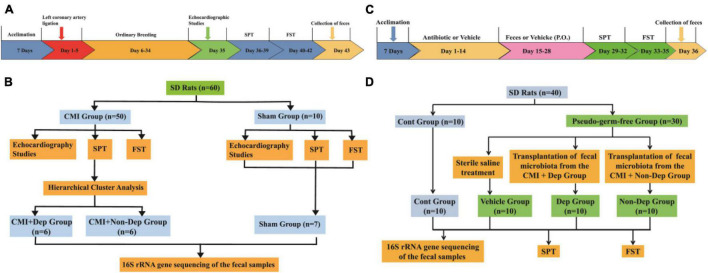
Experimental schedule. **(A)** The timeline of the first experiment. After acclimation, left coronary artery ligation was performed and then CMI models established after 4 weeks. Cardiac function was evaluated on day 35. SPT was performed on days 36–39. FST was performed on days 40–42. Fecal samples were collected on day 43. **(B)** The flow chart of the first experiment. Fifty male SD rats were randomly used to establish the CMI models and 10 to the sham models. **(C)** The timeline of the second experiment. After being acclimated to a new environment for 7 days, rats were randomly treated by giving them large doses of antibiotic solution for 14 consecutive days (days 1–14). Then, pseudo-germ-free rats were orally treated with fecal microbiota from CMI + Dep or CMI + Non-Dep group rats from days 15 to 28. Subsequently, SPT was measured on days 29–32. FST was performed on days 33–35. Fecal samples were collected for 16S rRNA gene sequencing testing on day 36. **(D)** The flow chart of the second experiment. Thirty male SD rats were randomly used to establish pseudo-germ-free rat models and 10 to the control group. Then the 30 pseudo-germ-free rats were randomly assigned to fecal microbiota transplantation. The fecal microbiota for transplantation was collected from the rats of the CMI + Dep and CMI + Non-Dep groups. CMI, chronic myocardial infarction; SPT, sucrose preference test; FST, forced swimming test; Dep, depression-like behavior; Non-Dep, non-depression-like behavior.

This study was divided into two parts. The first part of the study was designed to compare the difference of the gut microbiota between the Sham group, CMI + Dep group, and CMI + Non-Dep group. Fifty male SD rats were randomly used to establish the CMI models and 10 to the sham models. In the second part, 30 male SD rats were randomly used to establish pseudo-germ-free rat models and 10 to the control group. Then fecal microbiota transplantation was performed on the 30 pseudo-germ-free rats. The fecal microbiota for transplantation was collected from the CMI + Dep group and CMI + Non-Dep group rats. Detailed experimental schedule is shown in Figure 1.

### Chronic Myocardial Infarction Model

Left anterior descending coronary artery ligation was performed to produce a CMI rat model (Wang et al., 2013; Li, 2018). The rats fasted for 12 h and were deprived of water for 4 h before modeling. We used sodium pentobarbital (50–60 mg/kg intraperitoneal) as anesthetic and confirmed the adequacy of anesthesia by the absence of a withdrawal response to hindpaw nociceptive stimulation. Under sodium pentobarbital anesthesia and intubation, 2-cm incisions were made to the left of and parallel to the sterna in rats. Then, the third and fourth ribs were separated with a clamp, and the hearts were exposed. The pericardium was torn open, and the anterior descending branch of the coronary artery was ligated with 6/0 suture approximately 1–2 mm below the left auricle. Sham group rats underwent an identical surgery but did not sustain a left descending coronary artery ligation. Rats were monitored by electrocardiogram (ECG) before and after coronary artery ligation. Ischemia was confirmed by the myocardial surface becoming blanched, and ST–T segment elevation in ECG recordings was performed on the rats. After the surgery, lidocaine cream was administered to the wound locally twice daily for 3 days for incision pain. Four weeks later, we performed the following evaluations (Lin et al., 2009; Ito et al., 2013).

### Cardiac Function and Cardiac Morphology

Cardiac function was evaluated by using echocardiography (Lin et al., 2009; Ito et al., 2013). Under light sodium pentobarbital anesthesia with spontaneous respiration, we performed serial M-mode echocardiography by an echocardiography system (VividTM E95, French). We measured left ventricular internal diameter at end-diastole (LVIDd), left ventricular internal diameter at end-systolic (LVIDs), and left ventricular ejection fraction (LVEF), the posterior left ventricular (LV) wall, and the percent fractional shortening (%FS). The criterion of a successful CMI model was LVEF less than 60% and the percent fractional shortening (%FS) less than 30% (Zhang et al., 2010).

After all the experiments were done, rats were sacrificed, and their hearts were repeatedly rinsed with phosphate-buffered saline (PBS) buffer solution to remove the redundant tissues such as right ventricle, right atrium, and great vessels. Ventricular muscle tissues were taken and fixed with 4% paraformaldehyde solution. Samples were alcohol-dehydrated and paraffin-embedded, sections were made, and Masson staining was performed. Masson stain blue was used for collagen fibers and red for cardiomyocytes. The experimental process is shown in Figure 1.

### Behavioral Tests

Depression-like behavior was evaluated by SPT and FST. All tests were conducted individually, in the morning (between 9 and 11 a.m.). The detailed schedule is shown in Figure 1.

#### Sucrose Preference Test

This test consisted of a two-bottle procedure in which rats were given the choice between water and 1% sucrose solution. Rats were habituated to drink water from two bottles during days 1 and 2. At day 3, rats were deprived of water and food. At day 4, rats were exposed to two identical bottles again: one was water, and another was 1% sucrose solution. The bottles containing water and sucrose were weighed before and at the end of this period. The sucrose preference [sucrose consumption/(sucrose consumption + water consumption)] was used as a measure for depression-like behavior (Iniguez et al., 2014). The liquid used in the experiment was sterile.

#### Forced Swimming Test

In FST, rats were forced to swim under inescapable conditions. Initially, rats tried to escape but eventually adopted a static posture in which they make only the movements necessary to maintain their head above water. The immobility time was used as a measure for depression-like behavior. Specifically, all rats were placed individually in a clear plastic cylinder (diameter: 25 cm; height: 35 cm) containing 20 cm of water, maintained at 23 ± 1°C. Rats were forced to swim for five consecutive minutes, and immobility time was recorded (Iniguez et al., 2014; Yang et al., 2019).

### Pseudo-Germ-Free Rats Modeling

Forty male SD rats were acclimated to a new environment for 7 days. Thirty rats were randomly chosen to perform the pseudo-germ-free rat models and 10 to the control group. Broad-spectrum antibiotics [ampicillin 1 g/L, neomycin sulfate 1 g/L, metronidazole 1 g/L (Amresco Co., Ltd., United States)] were dissolved in drinking water and administered *ad libitum* to the pseudo-germ-free group rats for 14 consecutive days. At the same time, the control group rats were administered to drinking water *ad libitum*. All drinking water was sterile and renewed every day.

### Fecal Samples Collection

After finishing the behavioral tests, feces from each group of rats were collected individually. All rats were separately placed in a clean cage containing sterilized filter paper. Fecal samples were collected immediately after defecation in a sterilized centrifuge tube. The filter paper was replaced for each rat. Feces were stored in a −80°C freezer until analysis or transplantation (Yang et al., 2017).

### Fecal Microbiota Transplantation

Fecal microbiota was prepared by diluting 1 g of fecal sample obtained from CMI + Dep group rats in 10 ml of sterile saline. The fecal material was suspended, and 0.2 ml of the suspension was administered by gavage to each recipient pseudo-germ-free rats for 14 consecutive days (Ge et al., 2017). Fecal samples from the CMI + Dep in the first experiment were randomly mixed. Rats that received fecal microbiota transplantation of the CMI + Dep group were marked as the Dep group. Fecal microbiota transplantation of the CMI + Non-Dep groups was performed in the same way. And the rats that received fecal microbiota transplantation of the CMI + Non-Dep group were marked as the Non-Dep group. Subsequently, behavioral tests were measured, and fecal samples of the Dep and Non-Dep group rats were collected for 16S rRNA gene sequencing analysis. The experimental process is shown in Figure 1.

### 16S rRNA Gene Sequencing of Fecal Samples

16S rRNA gene is mainly composed of defensive zone and variable zone. The conserved region represents the affinity between the microbiome, and the variable region represents the difference. Among them, V3–V4 had good specificity and complete database information (Clarridge, 2004). In view of this, we chose to use regions V3–V4 for bacterial diversity analysis. Fecal samples were collected after all behavioral tests, placed in 1.5-ml tubes, snap frozen on dry ice, and stored at −80°C prior to 16s rRNA gene sequencing at Shanghai OE Biotech. Co., Ltd (Shanghai, China). We used Vsearch (Version 2.4.2) (Rognes et al., 2016) to classify operational taxonomic units (OTUs) according to 97% similarity of valid tags obtained from quality control and selected the sequence with the maximum abundance in each OTU as the representative sequence of this OTU. The RDP Classifier Naive Bayesian classification algorithm (Wang et al., 2007) was used to compare and annotate the representative sequence with the database to obtain the annotation information of OTUs. All sequences were classified using the National Center for Biotechnology Information BLAST and SILVA databases. Distance calculation, OTU clustering, rarefaction analysis, and estimator calculation (α-diversity and β-diversity) were performed using the MOTHUR program (Sun et al., 2016).

### Receiver Operating Characteristic Curve Analyses

Receiver operating characteristic (ROC) curves illustrated the diagnostic ability of a binary classifier system with the true positive rate (sensitivity) as the ordinate and the false positive rate (1-specificity) as the abscissa. The ROC curves were used to distinguish rats with CMI-induced depression-like behavior from all other rats. The value of the area under the curve (AUC) represents the accuracy of the diagnosis.

### Statistical Analysis

Data was presented as the mean ± standard error of the mean (SEM). All statistical analyses were performed using GraphPad Prism 8 (GraphPad Software, San Diego, CA, United States). For the hierarchical cluster analysis of SPT results to define CMI + Dep and CMI + Non-Dep groups, the data were first standardized as Z scores. Hierarchical cluster analysis was performed using Ward’s method and applying squared Euclidean distance as the distance measure. Correlation analysis was conducted using Pearson’s product-moment coefficient. The diagnostic cutoff value, sensitivity, specificity, and accuracy of each bacterium were determined using ROC curve analysis. Group means were compared by one-way analysis of variance (ANOVA), followed by *post hoc* Tukey’s tests for pair-wise comparisons. A *p* < 0.05 (two-tailed) was considered significant for all tests.

## Results

### Differences in Depression-Like Behaviors Among Control, CMI + Dep, and CMI + Non-Dep Rats

**FIGURE 2:**
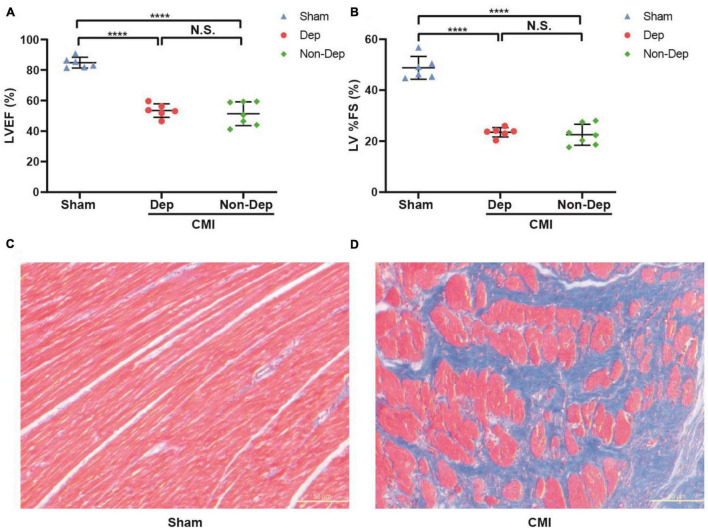
Cardiac function evaluated by echocardiography and comparisons of cardiac morphology between sham rats and CMI rats. **(A)** LVEF. **(B)** LV%FS. **(C)** Masson staining in the left ventricular cardiomyocytes extracted from sham rats. **(D)** Masson staining in the left ventricular cardiomyocytes extracted from CMI rats. ****p < 0.0001; N.S., not significant. Data are shown as mean ± SEM. (*n* = 6–7) by one-way ANOVA. CMI, chronic myocardial infarction; Dep, depression-like behavior; Non-Dep, non-depression-like behavior.

**FIGURE 3:**
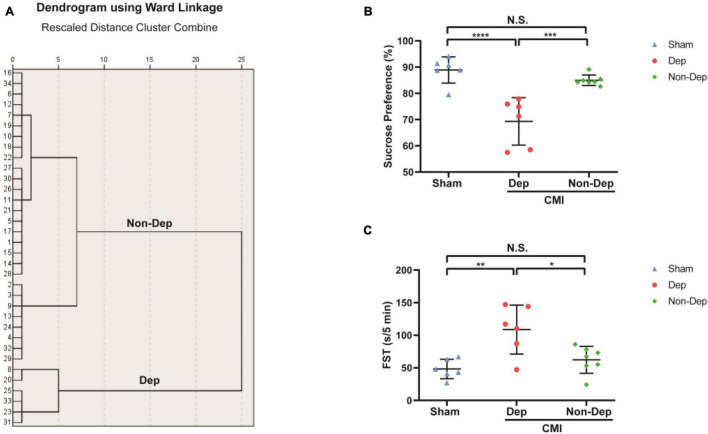
Comparisons of SPT and FST among Sham group, CMI + Dep group, and CMI + Non-Dep group rats. **(A)** Dendrogram of hierarchical clustering analysis. A total of 34 CMI rats were included, and 6 CMI rats were divided into depression-like behavior groups by SPT results of hierarchical clustering analysis. **(B)** SPT. **(C)** FST. Data are shown as mean ± SEM. (*n* = 6–7) by one-way ANOVA. *p < 0.05, **p < 0.01, ***p < 0.001, or ****p < 0.0001; N.S., not significant. CMI, chronic myocardial infarction; SPT, sucrose preference test; FST, forced swimming test; Dep, depression-like behavior; Non-Dep, non-depression-like behavior.

Echocardiography revealed that LVEF and %FS were both significantly greater in Sham rats than in CMI rats (84.83 ± 3.22% vs. 52.35 ± 6.06%, 48.77 ± 4.2% vs. 22.97 ± 3.04%, *p* < 0.05; Figures 2A,B). In the Sham group, Masson staining results showed that left ventricular myocardial cell aligned neatly with no obvious pathological changes. But the CMI group rats’ left ventricular myocardial cells were arranged in a disorderly manner, shapes were irregular, and there were a lot of blue collagen fibers and extensive fibrosis between myocardial cells (Figures 2C,D). All of these indicated the success of CMI modeling. And then CMI rats were divided into depression susceptible (CMI + Dep) and depression unsusceptible (CMI + Non-Dep) groups according to the results of hierarchical cluster analysis of SPT (Figure 3A). Compared to the Sham and CMI + Non-Dep groups, the immobility times of FST were significantly increased in CMI + Dep group (48.17 ± 13.55 s vs. 62.29 ± 19.01 s vs. 108.67 ± 34.31 s, *p* < 0.05; Figure 3C). And in SPT, sucrose preference was significantly decreased in CMI + Dep group but not in the sham or CMI + Non-Dep groups (69.33 ± 8.25 % vs. 88.90 ± 4.56% vs. 85.00 ± 1.85%, *p* < 0.05; Figure 3B). Therefore, CMI + Dep rats showed depression-like behavior.

### Differences in Gut Microbiota Profile Among Control, CMI + Dep, and CMI + Non-Dep Rats

**FIGURE 4:**
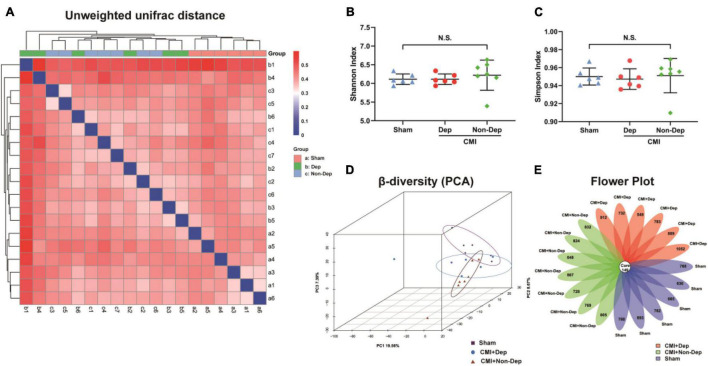
Differences in gut microbiota profiles among Sham, CMI + Dep, and CMI + Non-Dep group rats. **(A)** Unweighted unifrac diversity distance. **(B)** Shannon index (one-way ANOVA; F2, 16 = 0.3482, *p* > 0.05). **(C)** Simpson index (one-way ANOVA; F2, 16 = 0.1202, *p* > 0.05). **(D)** PCA analysis of gut microbiota. **(E)** Flower plot of OTUs distribution. The numbers in the core represented the OTUs shared by all samples, and the numbers on the petals represented the total OTUs of each sample minus the number of OTUs shared. The α-diversity was shown as mean ± SEM. (*n* = 6–7 individual fecal samples/group). N.S., not significant. CMI, chronic myocardial infarction; Dep, depression-like behavior; Non-Dep, non-depression-like behavior; PCA, principal component analysis.

We used 16S rRNA gene sequencing to determine alterations in gut microbiota composition among the Sham group, the CMI + Dep group, and the CMI + Non-Dep group rats after left coronary artery ligation. After quality control, the data volume of clean tags was distributed between 38,106 and 41,514. Valid tags were obtained by removing chimerism from clean tags and were finally used for analysis. The data volume of valid tags was distributed between 34,129 and 37,599, and the average length ranged from 429.12 to 433.94 bp. In the first experiment, the number of OTU in each sample ranged from 698 to 1,201 (Figure 4E). The number of differential OTUs was 100, and the number of differential genera was 10. Differential profiles of gut microbiota existed among the three groups. Unweighted unifrac diversity distance suggested remarkable differences in gut microbiota among the three groups (Figure 4A). Although Shannon and Simpson indices failed to show such a difference (Figures 4B,C), three-dimensional data of principal component analysis (PCA) revealed separated positions among groups (Figure 4D). PCA was a visualization method to make a comparison between sample diversity. In three-dimensional data of PCA, each dot represented a sample, and similar samples would be clustered together. Most of the dots from the sham, CMI + Dep, and CMI + Non-Dep groups were aggregated separately (Figure 4D).

### Differential Levels of Gut Microbiota Among Sham, CMI + Dep, and CMI + Non-Dep Rats

**FIGURE 5:**
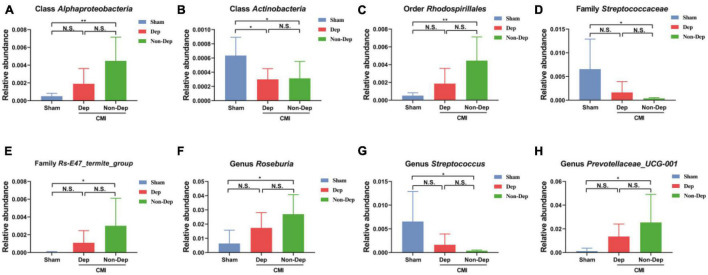
Differences in the relative abundance of various gut microbes among the Sham group, the CMI + Dep group, and the CMI + Non-Dep group. **(A)** Class *Alphaproteobacteria* (F2, 16 = 7.358, *p* < 0.05). **(B)** Class *Actinobacteria* (F2, 16 = 4.512, *p* < 0.05). **(C)** Order *Rhodospirillales* (F2, 16 = 7.270, *p* < 0.05). **(D)** Family *Streptococcaceae* (F2, 16 = 4.685, *p* < 0.05). **(E)** Family Rs-E47_termite_group (F2, 16 = 3.540, *p* < 0.05). **(F)** Genus *Roseburia* (F2, 16 = 5.120, *p* < 0.05). **(G)** Genus *Streptococcus* (F2, 16 = 4.685, *p* < 0.05). **(H)** Genus *Prevotellaceae*_UCG-001 (F2, 16 = 3.831, *p* < 0.05). Data are shown as mean ± SEM. (*n* = 6–7) by one-way ANOVA. CMI, chronic myocardial infarction; Dep, depression-like behavior; Non-Dep, non-depression-like behavior. *p < 0.05, **p < 0.01, N.S., not significant.

16S rRNA gene sequencing revealed that a total of eight gut bacteria at six phylogenetic levels (phylum, class, order, family, genus, and species) significantly differed among the Sham group, the CMI + Dep group, and the CMI + Non-Dep group (Figures 5A–H). At the class level, the relative abundances of *Actinobacteria* were significantly lower in the CMI + Dep group than Sham group (0.000299 ± 0.000138 vs. 0.000635 ± 0.000234, *p* < 0.05; Figure 5B).

### Correlations Between Depression-Like Behaviors and Specific Gut Bacteria

**FIGURE 6:**
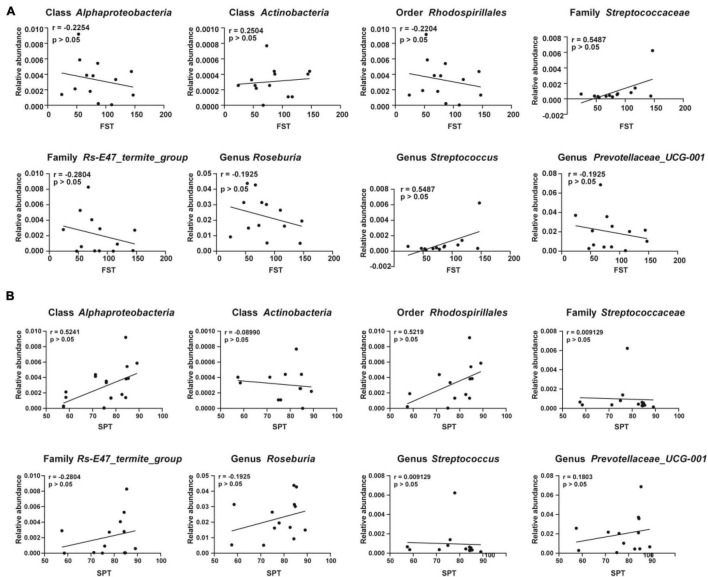
Correlations between the relative abundance of various gut microbes and the depression-like behavior. **(A)** Correlation analyses between the relative abundance of gut microbiota and the immobility time of FST. **(B)** Correlation analyses between the relative abundance of gut microbiota and the value of SPT. Significant correlations were determined based on *p* < 0.05. FST, forced swimming test; SPT, sucrose preference test.

Sucrose preference test and FST were adopted as measures to evaluate the effects of gut microbiota composition on depression-like behaviors. Correlations between the sucrose preference percentage of the CMI + Dep group and the CMI + Non-Dep group and the relative abundance of eight bacteria were analyzed. Correlations between the immobility time and the relative abundance of eight bacteria were also analyzed. Although the results were not statistically significant, they showed clear trends in the influence of gut microbiota on depression-like behaviors. The relative abundance of class *Alphaproteobacteria*, order *Rhodospirillales*, family *Rs-E47_termite_group*, and genus *Roseburia* negatively influenced depression-like behavior in rats (Figures 6A,B).

### Evaluation of Gut Bacteria for the Diagnosis of Chronic Myocardial Infarction-Induced Dep Using Receiver Operating Characteristic Curve Analysis

**FIGURE 7:**
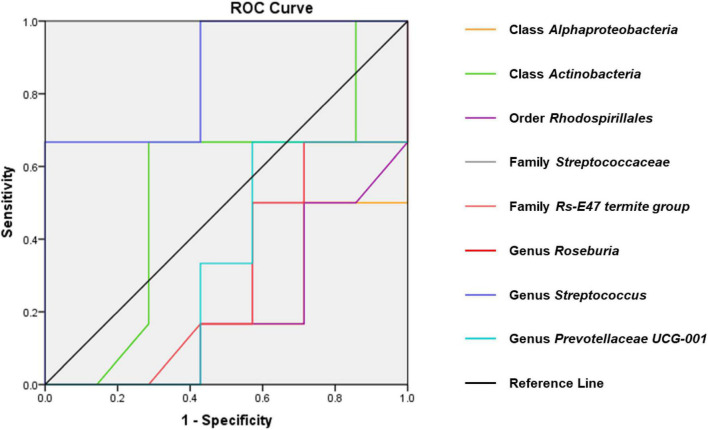
ROC curves of various gut microbiota for the depression-like behavior phenotypes of CMI rats. Class *Alphaproteobacteria* (AUC = 0.810). Class *Actinobacteria* (AUC = 0.536). Order *Rhodospirillales* (AUC = 0.798). Family *Streptococcaceae* (AUC = 0.857). Family *Rs-E47_termite_group* (AUC = 0.714). Genus *Roseburia* (AUC = 0.702). Genus *Streptococcus* (AUC = 0.667). Genus *Prevotellaceae_UCG-001* (AUC = 0.667). ROC, receiver operating characteristic; AUC, area under the curve.

Receiver operating characteristic curves were constructed to evaluate the diagnostic ability of the gut bacteria in depression (Figure 7). The best cutoff values, sensitivity, specificity, and accuracy as well as the positive and negative predictive values of gut bacteria for the diagnosis of CMI-induced Dep are summarized in Table 1.

**TABLE 1:** Evaluation of various gut microbiota for diagnosis of depression-like behavior.

Evaluation index	Cut-off value	Sensitivity	Specificity	Positive predictive value	Negative predictive value	Accuracy
Class *Alphaproteobacteria* (n)	0.003332967	83.33% (5/6)	71.43% (5/7)	71.43% (5/7)	83.33% (5/6)	76.93% (10/13)
Class *Actinobacteria* (n)	0.000256382	50% (3/6)	71.43% (5/7)	66.67% (4/6)	71.43% (5/7)	69.23% (9/13)
Order *Rhodospirillales* (n)	0.003332967	83.33% (5/6)	71.43% (5/7)	71.43% (5/7)	83.33% (5/6)	76.92% (10/13)
Family *Streptococcaceae* (n)	0.000622642	66.67% (4/6)	100% (7/7)	100% (4/4)	77.78% (7/9)	84.62% (11/13)
Family *Rs-E47 termite group* (n)	0.002893455	100% (6/6)	42.86% (3/7)	60% (6/10)	100% (3/3)	69.23% (9/13)
Genus *Roseburia* (n)	0.026480607	83.33% (5/6)	57.14% (4/7)	62.50% (5/8)	80% (4/5)	69.23% (9/13)
Genus *Streptococcus* (n)	0.000622642	66.67% (4/6)	100% (7/7)	100% (4/4)	77.78% (7/9)	84.62% (11/13)
Genus *Prevotellaceae UCG-001* (n)	0.025674834	100% (6/6)	42.86% (3/7)	60% (6/10)	100% (3/3)	69.23% (9/13)

### Effects of CMI + Dep and CMI + Non-Dep Gut Microbiota Transplantation on Depression-Like Behaviors in Pseudo-Germ-Free Rats

**FIGURE 8:**
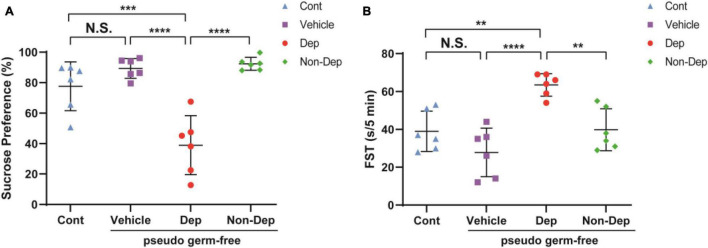
Effects of fecal microbiota transplantation on depression-like behaviors in pseudo-germ-free rats. **(A)** SPT. **(B)** FST. Data are shown as mean ± SEM. (*n* = 6) by one-way ANOVA, **p < 0.01, ***p < 0.001, or ****p < 0.0001; N.S., not significant. SPT, sucrose preference test; FST, forced swimming test; Cont, control; Dep, depression-like behavior; Non-Dep, non-depression-like behavior.

Pseudo-germ-free models were established by administering antibiotics for 14 days. Gut microbiota from CMI + Dep group and CMI + Non-Dep group rats were transplanted into the gastrointestinal tract of pseudo-germ-free rats through feces for another 14 consecutive days (Figure 1D). FST and SPT were performed on days 29–33 (Figure 1C). Compared to the control, vehicle, and Non-Dep group, sucrose preference percentage obviously decreased in Dep group, and the immobility time of FST also significantly increased (77.61 ± 14.63% vs. 89.36 ± 5.95% vs. 92.34 ± 3.88% vs. 38.95 ± 17.76%, 39.00 ± 9.68 s vs. 27.83 ± 11.72 s vs. 39.83 ± 10.09 s vs. 63.50 ± 5.44 s, *p* < 0.05, Figures 8A,B). Therefore, gut microbiota from CMI + Dep rats appeared to induce depression-like behavior in pseudo-germ-free rats.

### Differences in Gut Microbiota Composition Between Pseudo-Germ-Free Rats Receiving CMI + Dep or CMI + Non-Dep Rats Fecal Bacteria

**FIGURE 9:**
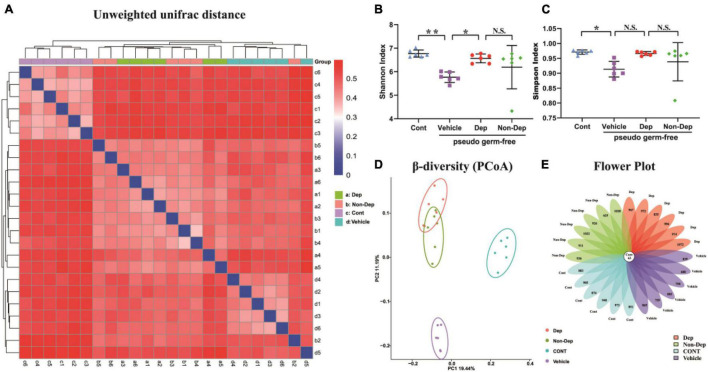
Differences in gut microbiota profiles among Cont, Vehicle, Dep, and Non-Dep rats. **(A)** Unweighted unifrac diversity distance. **(B)** Shannon index (one-way ANOVA; F3, 20 = 4.901, *p* < 0.05). **(C)** Simpson index (one-way ANOVA; F3, 20 = 3.503, *p* < 0.05). **(D)** PCoA analysis of gut microbiota. **(E)** Flower plot of OTUs distribution. The numbers in the core represent the OTUs shared by all samples, and the numbers on the petals represent the total OTUs of each sample minus the number of OTUs shared. The α-diversity is shown as mean ± SEM. (*n* = 6). *p < 0.05, **p < 0.01; N.S., not significant. Cont, control; Dep, depression-like behavior; Non-Dep, non-depression-like behavior; PCoA, principal coordinates analysis.

16S rRNA gene sequencing result showed that the number of OTUs ranged from 688 to 1,135. A flower plot showed that gut microbiota in the four groups were distinct (Figure 9E). There were 625 different OTUs, 57 different genera, and 6 different phyla. A plot of unweighted unifrac diversity distance demonstrated notable differences in gut microbiota composition among control pseudo-germ-free rats and those receiving vehicle, CMI + Dep rats fecal bacteria, or CMI + Non-Dep rats fecal bacteria (Figure 9A). α-Diversity refers to the diversity of species and bacteria within a community or habitat. Shannon as well as Simpson indices are commonly used to evaluate the α-diversity of gut microbiota (Zhan et al., 2018). Dep group remarkably restored the decrease of Shannon index compared to vehicle groups (6.56 ± 0.17 vs. 5.77 ± 0.20, *p* < 0.05, Figure 9B), whereas there was no significant change in Simpson index between Dep groups and vehicle groups (Figure 9C). β-Diversity using principal coordinates analysis (PCoA) demonstrated the differentiation among habitats (Guida et al., 2018). Clearly, the dots of the control group were individually separated from the others. And the dots of Dep and Non-Dep groups were also far away from the vehicle group. Compared with Non-Dep group, the composition of gut microbiota in Dep group showed a different profile (Figure 9D). Therefore, it was extremely possible that the composition of the gut microbiota was significantly different between control, vehicle, Dep, and Non-Dep groups.

### Differential Levels of Gut Microbiota in Pseudo-Germ-Free Rats

**FIGURE 10:**
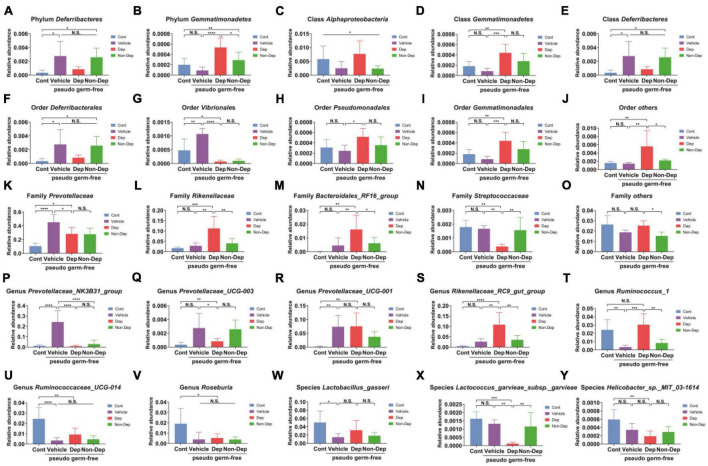
Differences in the relative abundance of various gut microbes among Cont, Vehicle, Dep, and Non-Dep rats. **(A)** Phylum *Deferribacteres* (F3, 20 = 5.505, *p* < 0.05). **(B)** Phylum *Gemmatimonadetes* (F3, 20 = 11.48, *p* < 0.05). **(C)** Class *Alphaproteobacteria* (F3, 20 = 3.181, *p* < 0.05). **(D)** Class *Gemmatimonadetes* (F3, 20 = 9.061, *p* < 0.05). **(E)** Class *Deferribacteres* (F3, 20 = 5.505, *p* < 0.05). **(F)** Order *Deferribacterales* (F3, 20 = 5.505, *p* < 0.05). **(G)** Order *Vibrionales* (F3, 20 = 23.98, *p* < 0.05). **(H)** Order *Pseudomonadales* (F3, 20 = 3.670, *p* < 0.05). **(I)** Order *Gemmatimonadales* (F3, 20 = 9.061, *p* < 0.05). **(J)** Other orders (F3, 20 = 6.168, *p* < 0.05). **(K)** Family *Prevotellaceae* (F3, 20 = 15.32, *p* < 0.05). **(L)** Family *Rikenellaceae* (F3, 20 = 10.79, *p* < 0.05). **(M)** Family *Bacteroidales_RF16_group* (F3, 20 = 6.924, *p* < 0.05). **(N)** Family *Streptococcaceae* (F3, 20 = 8.771, *p* < 0.05). **(O)** Other families (F3, 20 = 5.736, *p* < 0.05). **(P)** Genus *Prevotellaceae_NK3B31_group* (F3, 20 = 22.83, *p* < 0.05). **(Q)** Genus *Prevotellaceae_UCG-003* (F3, 20 = 5.505, *p* < 0.05). **(R)** Genus *Prevotellaceae_UCG-001* (F3, 20 = 6.673, *p* < 0.05). **(S)** Genus *Rikenellaceae_RC9_gut_group* (F3, 20 = 12.28, <0.05). **(T)** Genus *Ruminococcus_1* (F3, 20 = 11.46, *p* < 0.05). **(U)** Genus *Ruminococcaceae_UCG-014* (F3, 20 = 12.73, *p* < 0.05). **(V)** Genus *Roseburia* (F3, 20 = 4.602, *p* < 0.05). **(W)** Species *Lactobacillus_gasseri* (F3, 20 = 4.269, *p* < 0.05). **(X)** Species *Lactococcus_garvieae_subsp._garvieae* (F3, 20 = 10.62, *p* < 0.05). **(Y)** Species *Helicobacter_sp._MIT_03-1614* (F3, 20 = 6.308, *p* < 0.05). Data are shown as mean ± SEM. (*n* = 6–7) by one-way ANOVA. CMI, chronic myocardial infarction; Dep, depression-like behavior; Non-Dep, non-depression-like behavior. *p < 0.05, **p < 0.01, ***p < 0.001, or ****p < 0.0001; N.S., not significant.

Fecal transplantation significantly altered the relative abundances of gut microbiota in Dep and Non-Dep rats. At six levels, a total of 25 gut bacteria in the fecal samples had substantial differences among control rats and rats receiving vehicle, Dep rats fecal bacteria, or Non-Dep rats fecal bacteria (Figure 10). Compared to the Non-Dep group, the relative abundance of the phylum *Gemmatimonadetes*, the other orders, the family *Rikenellaceae*, the family *Bacteroidales RF16 group*, the other families, the genus *Rikenellaceae_RC9_gut_group*, and the genus *Ruminococcus_1* significantly increased in Dep group (Figures 10B,J,L,M,O,S,T), whereas the relative abundance of the family *Streptococcaceae* and the species *Lactococcus_garvieae_subsp._garvieae* decreased in Dep group (Figures 10N,X).

## Discussion

Left anterior descending coronary artery ligation has been commonly used to create myocardial infarction models (Wang et al., 2013; Li, 2018). In the present study, results of cardiac function and cardiac morphology suggested successful creation of CMI models. In humans, depression is a common psychiatric symptom of CMI and is an independent risk factor for disease prognosis. A higher risk of developing depression in patients with CMI suggests a shared pathogenesis, although the mechanisms remain unknown (Penninx et al., 2001). The SPT and FST could well assess the symptoms of depression-like behavior in rodents. And several lines of evidence observed depression symptoms in rodents approximately 4 weeks after left anterior descending coronary artery ligation (Lin et al., 2009; Ito et al., 2013). In this study, CMI rats could be stratified into CMI + Dep and CMI + Non-Dep groups by using the hierarchical cluster analysis of SPT results. The CMI + Dep rats also demonstrated longer immobility time in FST compared with both sham-operated rats and CMI + Non-Dep rats.

Gut microbiota diversity has been strongly associated with mood-relating behaviors, such as depression (Lin et al., 2009; Winter et al., 2018). Accumulating evidences indicated that gut microbiota influenced central neurochemistry and behavior, which is called the microbiota–gut–brain axis (Cryan and O’Mahony, 2011; Dinan and Cryan, 2017). In the present study, we observed no significant difference in α-diversity (consisting of Shannon and Simpson indices) among the Sham, CMI + Dep, and CMI + Non-Dep groups. However, the separation of groups according to β-diversity (PCA) suggested that the microbiota composition was significantly altered by the comorbidity of CMI and depression-like behavior. To the best of our knowledge, this was the first study to demonstrate the role of gut microbiota in individual differences of depression-like phenotype in rats with CMI. Additionally, this was also the first study adopting hierarchical cluster analysis to study the comorbidity of CMI and depression-like phenotypes in rodents.

In the present results, gut microbiota was significantly altered in the CMI + Dep or CMI + Non-Dep rats compared to sham-operated rats, suggesting a role of gut microbiota in the individual differences of depression-like phenotype. Furthermore, transplantation of fecal microbiota from CMI + Dep rats induced depression-like behavior in the antibiotics-treated pseudo-germ-free rats. However, transplantation of fecal microbiota from Non-Dep rats showed no tendency to depression-like behavior in the antibiotic-treated pseudo-germ-free rats, compared to the control group. Distinct gut microbial compositions were identified following fecal microbiota transplant. Taken all together, these results suggest that change in gut microbiota played an important role in the depression-like phenotypes in rats with CMI.

*Streptococcus* accounted for a large amount of deaths worldwide through diverse disease manifestations. Cardiac injury and life-threatening acute cardiac complications are more common in *Streptococcus* infection, compared to other bacterial infections (Alhamdi et al., 2015). Intriguingly, *Streptococcus* was recently reported to be higher in depression patients compared to healthy people (Barandouzi et al., 2020). This result was consistent with our findings that the phylum *Streptococcus* and the genus *Streptococcus* were enriched in the fecal samples of Dep rats. Therefore, *Streptococcus* infection was possibly associated with the onset and symptom of depression-like behavior in CMI rats.

Our study revealed significant inconsistencies in the abundance of *Rikenellaceae*, *Bacteroidales_RF16_group*, *Ruminococcus_1*, and *Lactococcus_garvieae_subsp._garvieae*. High abundance in *Rikenellaceae*, *Bacteroidales_RF16_group*, and *Ruminococcus_1* was observed in antibiotics-treated pseudo-germ-free rats treated with fecal microbiota from CMI + Dep rats. In contrast, *Lactococcus_garvieae_subsp._garvieae* observably decreased in antibiotics-treated pseudo-germ-free rats treated with CMI + Dep rats’ fecal microbiota, compared to the other groups. Although no study has reported on the role of these gut microbiota in depression, there were similar trends in *Bacteroidales* and *Lactococcus* in depression patients. Ma et al. found that the alterations of *Ruminococcus* were closely related to abnormalities of depression symptoms and inflammatory cytokines. *Rikenellaceae* correlated with butyric and valeric acids (Qing et al., 2019), and short-chain fatty acids (SCFAs) are regulatory compounds with the potential to influence inflammatory as well as emotional state and cognition through the gut–brain axis (Skonieczna-Zydecka et al., 2018). Nonetheless, further study was needed to clarify the role of *Rikenellaceae*, *Bacteroidales*, *Lactococcus*, and/or *Ruminococcus* in depressed patients with CMI. Collectively, gut microbiota may play a role in these behavioral abnormalities in CMI rats.

There were some limitations in our study. First, we did not investigate the influence of sex difference in gut microbiota composition in CMI-related depression, although the previous studies showed a gender-dependent difference in the gut microbiota composition (Haro et al., 2016; Bridgewater et al., 2017). Secondly, we did not identify the relationship between the specific microbiota species and depression-like behaviors. If we can identify the specific microbiota that contribute to depression-like phenotypes in CMI rats, these gut microbiota would be novel therapeutic targets for depression and anhedonia in patients with neuropathic pain.

In conclusion, the CMI-induced depression-like behavior was notably associated with dysbiosis in gut microbiota, and change in specific gut bacteria may be involved in the pathogenesis of this comorbidity. In light of the links between depression and CMI, the regulation of gut microbiota may be a potential therapeutic target for depression. However, further detailed studies are clearly needed to validate the role of gut microbiota in the pathogenesis and therapeutic mechanisms of the depression-like behavior in CMI patients.

## Data Availability Statement

The raw data supporting the conclusions of this article will be made available by the authors, without undue reservation.

## Ethics Statement

The animal study was reviewed and approved by Renmin Hospital of Wuhan University.

## Author Contributions

All authors critically reviewed and approved the final version for submission.

## Conflict of Interest

The authors declare that the research was conducted in the absence of any commercial or financial relationships that could be construed as a potential conflict of interest.

## Publisher’s Note

All claims expressed in this article are solely those of the authors and do not necessarily represent those of their affiliated organizations, or those of the publisher, the editors and the reviewers. Any product that may be evaluated in this article, or claim that may be made by its manufacturer, is not guaranteed or endorsed by the publisher.
